# The Relationship between Alcohol Consumption and Gout: A Mendelian Randomization Study

**DOI:** 10.3390/genes13040557

**Published:** 2022-03-22

**Authors:** Ali Alamdar Shah Syed, Aamir Fahira, Qiangzhen Yang, Jianhua Chen, Zhiqiang Li, Haibing Chen, Yongyong Shi

**Affiliations:** 1 Bio-X Institutes, Key Laboratory for the Genetics of Developmental and Neuropsychiatric Disorders (Ministry of Education), Shanghai Jiao Tong University, 1954 Huashan Road, Shanghai 200030, China; alamdar@sjtu.edu.cn (A.A.S.S.); amiralizai@sjtu.edu.cn (A.F.); lg10is1@sjtu.edu.cn (Q.Y.); jianhua.chen@smhc.org.cn (J.C.); lizqsjtu@163.com (Z.L.); 2 Department of Endocrinology and Metabolism, Shanghai 10th People’s Hospital, School of Medicine, Tongji University, 301 Yanchang Road, Shanghai 200072, China

**Keywords:** gout, MR, alcohol, hyperuricemia

## Abstract

Gout is a disease that manifests itself after decades of following a high-purine diet, with excessive alcohol consumption assumed to be one of the main contributors to the development of the disease. This study performs a Mendelian randomization (MR) analysis to determine whether alcohol consumption causally affects the risk of developing both hyperuricemia and gout. The results indicate that genetically predicted drinks consumed per week have no causal effect on neither the risk of gout (*p* = 0.35), nor serum uric acid levels (*p* = 0.73). For MR analysis in the other direction, genetic risk of gout was significantly associated with drinks per week (*p* = 0.03). Furthermore, the results of the MR analysis were verified in a cohort of individuals diagnosed with hyperuricemia and gout, comprising of alcohol-consuming and alcohol-abstaining subgroups. When split by alcohol status, the serum uric acid levels failed to show a significant difference in both gout (*p* = 0.92) and hyperuricemia (*p* = 0.23) subgroups. Overall, the results suggest that increased alcohol consumption does not play a causal role in the development of gout.

## 1. Introduction

Gout is a type of inflammatory arthritis caused by the deposition of monosodium urate crystals onto different joints of the body, followed by an extremely painful immune response [1]. Initially termed the disease of kings, the incidence of gout is steadily on the rise globally, as rapid economic development has provided lower socioeconomic classes with access to a richer diet [2]. The prevalence of gout in developed countries varies greatly, with estimates ranging from 3.9% in the USA to 0.3% in the Czech Republic and Portugal [3,4,5]. High levels of serum uric acid are widely believed to be primarily responsible for gout, with depositions occurring once the levels of serum uric acid exceed the saturation capacity of the blood. Hyperuricemia is defined as having sustained serum uric acid concentration levels above 6.6 mg/dL [6], is the greatest risk factor for gout. Other risk factors include gender, with males displaying a higher incidence rate of gout at increasing rates as they age [7]. Rural populations show a lower rate of gout incidence compared with those living in urban areas [8,9]. Comorbidities of gout include metabolic syndrome, cardiovascular diseases and renal diseases, with obese individuals displaying a significantly different incidence of gout (11.8% vs. 1.9% in women aged 70 years) [10]. Overall individuals that suffer congestive heart failure and renal diseases show four- and six-fold increase in risk of gout [11].

Monosodium urate crystal deposition, as a result of hyperuricemia, is now universally considered the cause of gout [1], with the heritability of serum uric acid levels estimated at between 60% and 30% [12]. Genes with large effects on serum urate variability identified by genome-wide association studies (GWAS) and candidate gene studies include genes such as *ABCG2*, *SLC2A9* and *SLC22A12* [13,14,15], all of which are involved in urate transport. Even though the genetic heritability of serum urate levels is high, environmental factors such as diet also plays a large role in the development of hyperuricemia and gout. As mentioned above, for many centuries it was assumed that gout was caused by lavish lifestyle often adopted by the richest members of society [2]. This diet was assumed to be rich in meats—red meat as well as seafood—along with frequent consumption of alcohol.

It was not until recently that it was possible to conduct large scale cohort studies over long durations to determine whether these lifestyle choices do indeed increase the risk of gout. Results from these studies do show that more than 2 servings of red meat per week increases the risk of incidence of gout (RR = 1.50), this increasing risk of gout remains true in regard to other well-known dietary risk factors such as seafood (RR = 1.51) and alcohol (RR = 1.81) [11,16,17]. Furthermore, when it comes to alcohol, beer appears to be the greatest culprit (RR = 2.51), followed by spirits (RR = 1.60), while the consumption of wine only showed a mild increased in risk of gout incidence (RR = 1.05) [16]. A recent review of dietary recommendations by clinicians for gout patients revealed that the most common recommendation is to completely avoid or limit alcohol intake (88%), surprisingly more than even losing weight (71%) [18]. Considering the widespread consumption of alcohol worldwide, dietary recommendations to eliminate alcohol for gout patients and the ever-increasing evidence from observational studies, here we attempt to determine whether the associations between alcohol consumption and gout are truly causal.

Mendelian randomization is a powerful alternative to randomized control trials that employs logistic regression and genetic association data from GWAS to determine causal exposure–outcome relationships. Several mendelian randomization studies have been performed exploring the causal role of alcohol on several diseases [19,20,21], notable findings include higher alcohol consumption increases the risk of peripheral artery disease and stroke. Similarly, in the past mendelian randomization has been successfully employed to establish that serum uric acid does not have a causal effect on the risk of chronic kidney disease and that both testosterone and caffeine have a protective effect on the risk of gout [22,23,24]. In this study, we aimed to determine whether a causal relationship exists between alcohol and gout or hyperuricemia, and, if so, to identify the direction of the relationship. To the best of our knowledge, this is the first Mendelian randomization study investigating the relationship between alcohol and gout.

## 2. Methods

### 2.1. Exposure and Outcome Datasets

The latest GWAS datasets for the traits in question were sourced from the GWAS catalog and a manual search of the existing literature. For alcohol consumption, we used a recently published meta-analysis investigating substance abuse, which included 941,280 individuals and identified 99 variants associated with drinks per week (DPW) at a significant genome-wide level [25]. For serum uric acid levels and the risk of gout, the genetic associations were derived from trans-ancestry meta-analysis, with a sample size of 457,690 individuals for serum uric acid levels and 334,880 individuals for the risk of gout [26].

### 2.2. Mendelian Randomization Study Design

In order to perform a mendelian randomization (MR) analysis, the following conditions must be met: (1) the genetic variants included in the analysis must be strongly associated with the exposure of interest; (2) these variants should not be associated with confounders of the exposure–outcome relationship; (3) the included variants should only affect the outcome through the exposure.

In this study, we attempted to determine the causal effect of alcohol consumption on the risk of developing gout using two-sample MR. We first extracted genome-wide significant SNPs from the exposure GWAs (*p* < 5 × 10^−8^) in order to satisfy the first assumption. Furthermore, we excluded SNPs with high pairwise correlation to ensure that all SNPs included were completely independent of each other (LD threshold < 0.01). Lastly, the included SNPs underwent a harmonization process to ensure that the effect reported was for the same alleles.

To calculate the causal estimates, we employed the inverse variance weighted (IVW) model, which is the model with the greatest power to detect causal relationships. High serum uric acid levels are believed to be a causal factor in the incidence of gout; however, not all individuals with high serum uric acid levels develop gout. Therefore, we can say that serum uric acid levels are a confounder in relationship between alcohol consumption and gout. In order to validate the second assumption of MR (that the variants are not associated with confounders of exposure–outcome relationship), we also performed MR analysis of alcohol consumption with serum uric acid levels.

The third assumption of MR, that the genetic instrument only effects the outcome through the exposure, was assessed using the MR–Egger intercept method, which, unlike the IVW method, calculates the intercept of exposure–outcome relationship. If the MR–Egger intercept is significantly different from zero, there is evidence of directional pleiotropy, and the genetic instrument is invalid.

In addition to the MR–Egger method, we also used the weighted median and leave-one-out methods as sensitivity analyses. The weighted median method can compute the MR estimates if only half of the instrument is valid, while the leave-one-out analysis helps determine if any one SNP is having a disproportionate effect on whole analysis.

### 2.3. Sample Recruitment and Serum Uric Acid Analysis

To verify the results of our mendelian randomization analysis in a clinical setting, we recruited a total of 40 recently diagnosed hyperuricemic and gout patients, which were further divided according to alcohol status. The subject groups were as follows: non-alcoholic hyperuricemia (*n* = 10), alcoholic hyperuricemia (*n* = 10), non-alcoholic gout (*n* = 10), and alcoholic gout (*n* = 10); additionally, a group of 10 healthy, matched controls were also recruited. Subjects were excluded if they were: (a) below 18 years of age, (b) received urate-lowering therapy in the past, (c) female, or (d) suffered from cancers, kidney, or blood diseases. All participants provided written consent and the study was approved by the ethics committee of the Shanghai Jiao Tong University Affiliated Sixth People’s Hospital and was performed in accordance with the declaration of Helsinki.

Serum was extracted from the subjects and stored at −20 °C for LC-MS analysis under the following conditions: The metabolic profiles were acquired by using Q Exactive quadruple Orbitrap high-resolution mass spectrometer. Chromatographic separation of metabolites was performed using Ultimate 3000 UHPLC system (Thermo Scientific, San Jose, CA, USA). Measures of 5 μL and 2 μL aliquots of the reconstituted metabolite extracts were injected for Reversed-Phase (RP) and Hydrophilic Interaction Liquid Chromatography (HILIC) analyses, respectively. Mobile phases containing 0.1% formic acid–water and 0.1% formic acid–acetonitrile were utilized for RP separation with 15 min fast gradient. A binary mobile phase comprising of 15% water in acetonitrile containing 10 mM ammonium acetate with pH adjusted to 10 with ammonium hydroxide and 50% water in acetonitrile containing the same buffer salt was employed for HILIC analysis. The mass spectrometer was operated under electrospray positive and negative ionization mode for RP and HILIC separation, respectively. The ion source parameters were set as below: sheath gas 45 arb, aux gas 10 arb, heater temperature 350 °C (300 °C for HILIC), ion transfer capillary temperature 300 °C, S-lens voltage 50%. The 70~1000 *m/z* ionized metabolic features were profiled under 70,000 FWHM resolution with 250 ms maximum ion injection time and 1 × 10^6^ AGC target. The key settings of ddMS2 mode include: 17,500 FWHM resolution, 1 × 10^5^ AGC target, 1.0 Da precursor isolation window, loop count 3, stepped NCE 15%, 30% and 45%, dynamic 5~8 s. Additionally, the top 100 background ions in the solvent blank were put into the exclusion list to improve the ddMS2 quality.

### 2.4. Statistical Analysis

All statistical analyses were performed in R, using the TwoSampleMR (version 0.5.5) and the MendelianRandomization (version 0.5) packages. A Student’s *t*-test and ANOVA were used to determine the difference between two individual groups and all groups, respectively. *p*-values of <0.05 were considered to be significant.

## 3. Results

### 3.1. Mendelian Randomization

**Figure 1:**
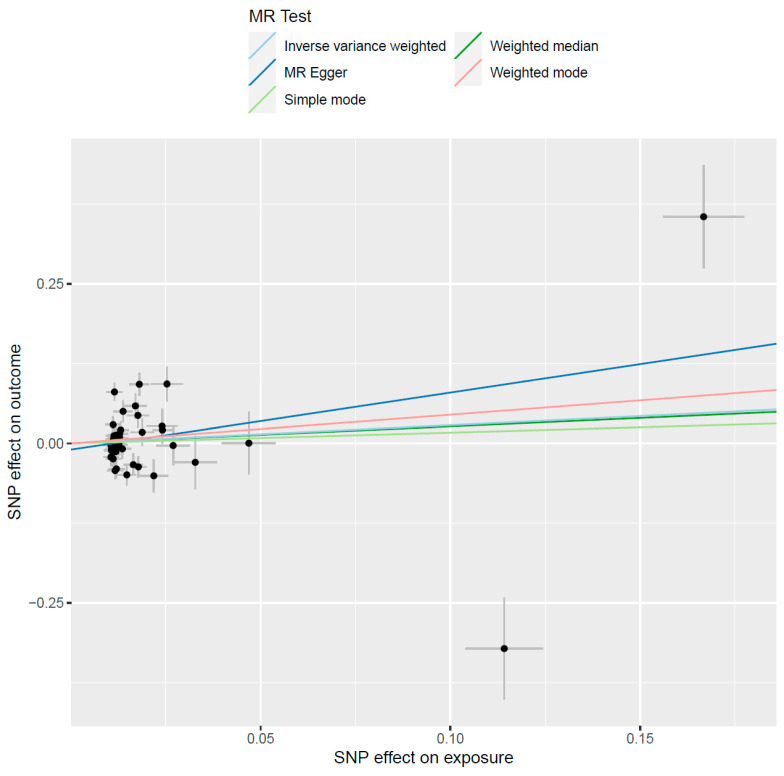
Causal effects of alcohol consumption on the risk of developing gout. The black circles represent the effect of individual SNPs on outcome and exposure, while the grey lines show their standard errors.

A summary of the MR results is shown in Table 1. After accounting for LD and the harmonization process, the total number of variants that were included in the MR investigating the effect of alcohol consumption on gout was 49. The results of this analysis showed that alcohol consumption does not cause gout (β = 0.28, se = 0.30, *p* = 0.35) (Figure 1), while the weighted median MR analysis also reported a similarly non-significant result (β = 0.26, se = 0.27, *p* = 0.33). The results from the MR–Egger intercept also showed no evidence of horizontal pleiotropy (intercept = −0.009, *p* = 0.35). The leave-one-out analysis failed to detect any SNPs that may have a disproportional effect on the result.

**Figure 2:**
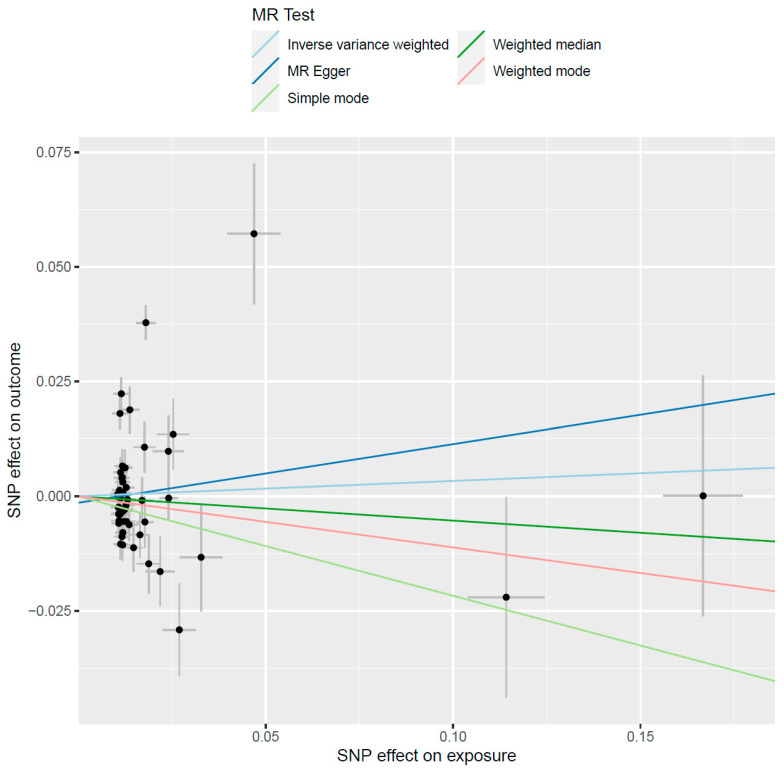
The causal effect of alcohol consumption on serum uric acid levels. The black circles represent the effect of individual SNPs on outcome and exposure, while the grey lines show their standard errors.

The total number of variants included in the genetic instrument the MR analysis investigating the causal effect of alcohol consumption on serum uric acid levels, which is a confounder of the alcohol-gout relationship, consisted of 50 SNPs and the MR analysis also resulted in non-significant estimates of the causal relationship (β = 0.03, se = 0.09, *p* = 0.73) (Figure 2). This result was consistent with the weighted median MR analysis, which yielded non-significant results as well (β = −0.05, se = 0.07, *p* = 0.44), while there was no evidence of horizontal pleiotropy (intercept = −0.001, *p* = 0.69). 

**Figure 3:**
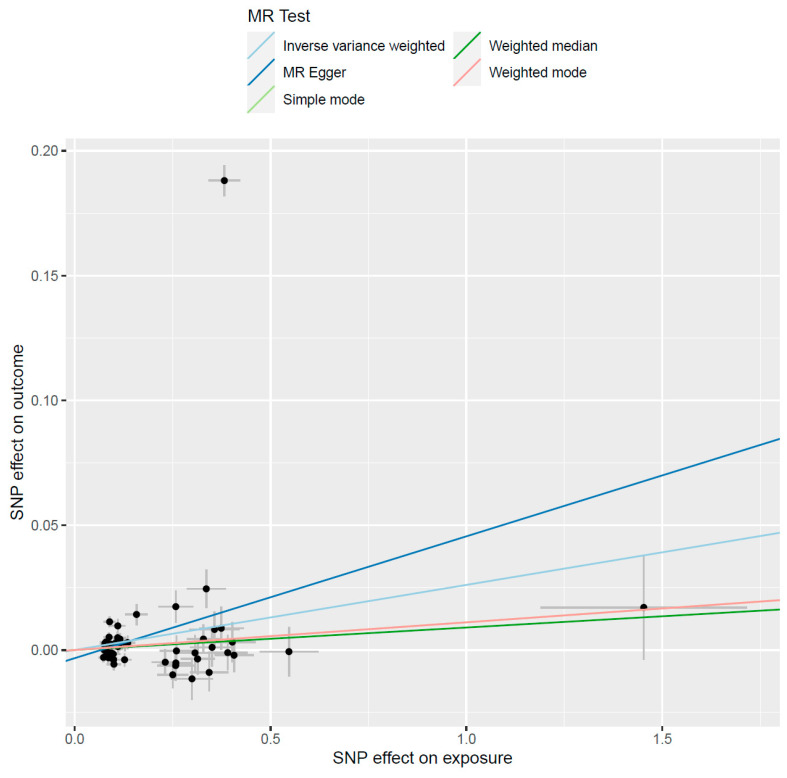
Causal effect of genetic risk of gout on alcohol consumption. The black circles represent the effect of individual SNPs on outcome and exposure, while the grey lines show their standard errors.

Interestingly the results from the MR in the other direction, a significant causal effect of risk of gout on alcohol consumption was observed (β = 0.026, se = 0.012, *p* = 0.037), with the weighted median MR sensitivity analysis displaying consistent results (β = 0.009, se = 0.004, *p* = 0.048) (Figure 3). Additionally, there was no sign of horizontal pleiotropy (intercept = −0.003, *p* = 0.29); however, the leave-one-out analysis showed that exclusion of variant rs2384575 changed the IVW *p* value to 0.053, which is only borderline significant. While the MR analysis investigating the effect genetically predicted serum uric acid levels on drinks per week failed to identify a significant causal relationship (β = 0.005, se = 0.007, *p* = 0.41). The weighted median MR results were also consistent with the main result (β = −0.001, se = 0.006, *p* = 0.79), while no evidence of horizontal pleiotropy was observed (intercept = 0.002, *p* = 0.56).

### 3.2. Serum Uric Acid Analysis

**Figure 4:**
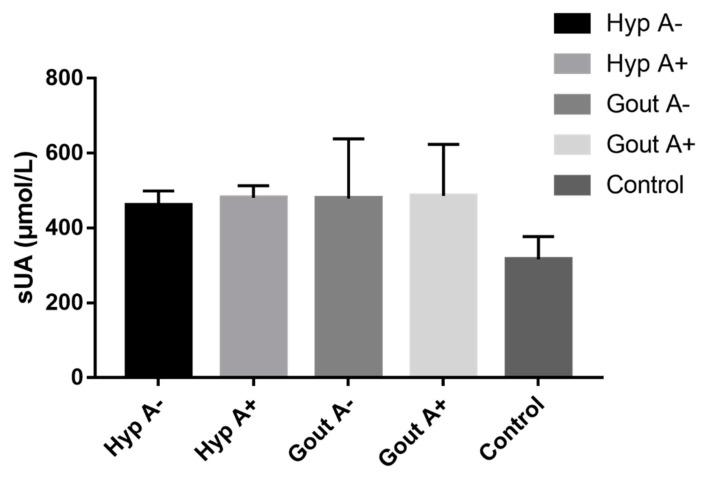
Serum uric acid levels between the different groups.

The characteristics of the patients enrolled in our serum uric acid analysis have been summarized in Table 2 and comparison of uric acid levels in different groups is shown in Figure 4. The results show that the highest levels of uric acid were observed in the alcoholic gout group, while the lowest levels were observed in healthy controls; however, there was no significant difference observed between alcohol consuming and alcohol abstaining groups. For gout, the mean serum uric acid level of the alcohol consuming group was 485.4 μmol/L vs. 479.3 μmol/L for the gout group that did not consume alcohol (*p* = 0.928), while for the hyperuricemic alcohol consuming group, the mean serum level of uric acid was 480.3 μmol/L vs. 460.7 μmol/L for the hyperuricemic alcohol abstaining group (*p* = 0.233). All hyperuricemic and gout groups had significantly higher serum uric acid levels compared with the healthy controls.

## 4. Discussion

In this study, we conducted a bidirectional mendelian randomization analysis to determine whether there is a causal relationship between alcohol consumption and serum uric acid levels or the risk of developing gout. Contrary to results from a meta-analysis which suggested that alcohol consumption increased the risk of hyperuricemia and gout [27], we found that the number of alcoholic beverages consumed per week had no causal effect on serum uric acid levels nor the risk of developing gout. Another mendelian randomization study conducted on Korean hyperuricemic individuals [28] reported that there was a significant causal relationship between increased alcohol consumption and the incidence of hyperuricemia; however, our results show that the number of drinks consumed per week do not have an casual effect on uric acid levels in the blood, which is different from the incidence of hyperuricemia. They went on to report that this was particularly true for men, while not achieving the significance threshold after accounting for multiple testing in women; however, their results are specific to the Korean population only.

Evidence from recent studies suggest that increased alcohol consumption does show an increased relative risk of gout [27]. In order to determine whether alcohol consumption significantly increased the serum uric acid levels of gout and hyperuricemic patients, we recruited a cohort of individuals suffering from gout and hyperuricemia and split them according to alcohol consumption status. We found that alcohol consumption did not significantly alter the serum uric acid levels in either of the groups, supporting the results from our mendelian randomization analysis. Since high, sustained levels of serum uric acid are a prerequisite to gout, we may conclusively say that alcohol consumption does not significantly alter serum uric acid levels and therefore may not play a causal role in development of hyperuricemia or gout. It must be noted that the alcoholic vs. non-alcoholic hyperuricemia group may have achieved some degree of significance if our sample size were larger, however; we consider we do not consider this to be the case in the gout group.

The genetics of hyperuricemia, which is universally accepted to be the cause of gout, involves genes such as *SLC2A9* (encodes for GLUT9), *SLC22A12* (encodes for URAT1), *SLC22A13* (encodes for OAT4) and *ABCG2*; these are mostly involved in uric acid reabsorption and secretion. Abnormally high levels of serum uric acid are usually caused by dysfunction of these transporter proteins, and it may be possible that alcohol consumption could exuberate serum uric acid levels but would be unable to do so independent of uric acid transporter dysfunction. However, beer contains purine guanosine, the breakdown of which would definitely result in an increase in serum uric acid levels when compared with other alcohol types [16].

According to our results, the genetically predicted risk of gout is causally associated with an increase genetically predicted risk of increased drinks consumed per week. In layman terms, individuals with gout are more likely to drink more alcohol and this may be the cause of the multiple associations between gout and alcohol use observed over the years; however, further research is required to reach a concrete conclusion on this matter.

A major limitation of our mendelian randomization analysis is that the dataset used for alcohol consumption (drinks per week), did not differentiate between the type of alcohol consumed. This is of importance since different alcohol types may confer different risk of gout development, which is particularly true for beer. Another limitation of our mendelian randomization analysis is that we could not stratify our dataset by gender; especially since gout is more prevalent in males than females, gender-specific analysis could bring further findings to light. Furthermore, the sample size of our serum uric acid analysis could have been higher, but we believe the sample size to be sufficient for the purposes of this study.

## 5. Conclusions

In summary, we performed a mendelian randomization study to investigate the causal relationships underlying the reported associations between alcohol consumption and hyperuricemia and gout. We found that alcohol consumption had no causal effect on the development of either hyperuricemia or gout, which was verified in an independent group of hyperuricemic and gout patients. Interestingly, according to our mendelian randomization analysis, increased risk of gout causes an increased in number of drinks consumed per week, suggesting that drinking alcohol does not cause gout, but rather those with gout are just more likely to consume alcohol more often. To the best of our knowledge, this is the first mendelian randomization study to investigate the causal relationship between alcohol and gout and the first to find evidence that contrary to popular belief the relationship between alcohol consumption and gout. Further research into the relationship between different alcohol types, with an emphasis on beer, and gout would be required to better understand the nature and direction of this relationship. Our work has provided insights that open avenues for future research and may aid clinicians make better recommendations as well as help gout patients make more informed life decisions.

## Figures and Tables

**Table 1:** Summary of results from the Mendelian randomization analyses. The main analysis was performed using IVW—inverse variance weighted mendelian randomization analysis (values shown are β-coefficients). Sensitivity analyses were performed: WM—weighted median; MR–Egger mendelian randomization methods.

Exposure	Outcome	n SNPs	IVW(se)	*p*-Value	WM-*p* Value	MR–Egger–*p*-Value	MR–Egger Intercept (*p*-Value)
DPW	Gout	49	0.285 (0.30)	0.35	0.31	0.21	0.35
DPW	SUA	50	0.033 (0.09)	0.73	0.44	0.62	0.695
Gout	DPW	56	0.026 (0.125)	**0.037**	**0.048**	0.053	0.29

**Table 2:** Characteristics of individuals included in the serum uric acid analysis, −/+ refer to the alcohol consumption status, for example Hpy A− would refer to the group with hyperuricemia which does not consume in alcohol.

Group	Hyp A−	Hyp A+	Gout A−	Gout A+	Control
Age (year)	45.1 (13.84)	49.5 (8.61)	41.8 (13.53)	53.0 (5.89)	41.8 (13.53)
Duration of Hyperuricemia (year)	0.9 (1.45)	2.4 (4.20)	12.89 (7.82)	11.2 (3.83)	0
Duration of gout (year)	/	/	11.70 (8.15)	11.56 (3.58)	/
Duration of drinking(year)	/	22.0 (8.882)	/	27.67 (13.88)	/
sUA (μmol/L)	460.7 (38.57)	480.30 (32.43)	479.3 (158.91)	485.4 (138.19)	316.2 (60.88)
Tophus (%)	0	0	6 (60%)	2 (20%)	/
Hypertension (%)	4 (40%)	3 (30%)	6 (60%)	4 (40%)	/
Diabetes (%)	0	0	1 (10%)	2 (20%)	/
Coronary heart disease (%)	0	0	0	0	/
Hyperlipidemia (%)	1 (10%)	0	6 (60%)	4 (40%)	/

## Data Availability

The mendelian randomization study was performed using publicly available data that can be found at https://www.ebi.ac.uk/gwas/ (accessed date 13 January 2022), or by extraction from the references provided in the methods section describing the exposure and outcome datasets.
